# Solubility and Permeation of Hydrogen Sulfide in Lipid Membranes

**DOI:** 10.1371/journal.pone.0034562

**Published:** 2012-04-11

**Authors:** Ernesto Cuevasanta, Ana Denicola, Beatriz Alvarez, Matías N. Möller

**Affiliations:** 1 Laboratorio de Enzimología, Facultad de Ciencias, Universidad de la República, Montevideo, Uruguay; 2 Center for Free Radical and Biomedical Research, Universidad de la República, Montevideo, Uruguay; 3 Laboratorio de Fisicoquímica Biológica, Facultad de Ciencias, Universidad de la República, Montevideo, Uruguay; Pennington Biomedical Research Center, United States of America

## Abstract

Hydrogen sulfide (H_2_S) is mainly known for its toxicity but has recently been shown to be produced endogenously in mammalian tissues and to be associated with physiological regulatory functions. To better understand the role of biomembranes in modulating its biological distribution and effects; we measured the partition coefficient of H_2_S in models of biological membranes. The partition coefficients were found to be 2.1±0.2, 1.9±0.5 and 2.0±0.6 in *n*-octanol, hexane and dilauroylphosphatidylcholine liposome membranes relative to water, respectively (25°C). This two-fold higher concentration of H_2_S in the membrane translates into a rapid membrane permeability, P_m_ = 3 cm s^−1^. We used a mathematical model in three dimensions to gain insight into the diffusion of total sulfide in tissues. This model shows that the sphere of action of sulfide produced by a single cell expands to involve more than 200 neighboring cells, and that the resistance imposed by lipid membranes has a significant effect on the diffusional spread of sulfide at pH 7.4, increasing local concentrations. These results support the role of hydrogen sulfide as a paracrine signaling molecule and reveal advantageous pharmacokinetic properties for its therapeutic applications.

## Introduction

For years, the toxicity of hydrogen sulfide (H_2_S, IUPAC recommended name dihydrogen sulfide) has been recognized and explored [1], but only recently it has been associated with an intrinsic physiological role in mammals. The discovery of endogenous H_2_S-producing enzymatic pathways and the measurement of significant H_2_S levels in several tissues was followed by the implication of H_2_S in numerous biochemical functions, and latest investigations propose that H_2_S could play signaling and cytoprotective roles, revealing its potential for pharmacological applications [2].

Hydrogen sulfide is a secondary product of the transsulfuration pathway. It is produced by two pyridoxal-phosphate-dependent enzymes, cystathionine β-synthase (CBS) [3] and cystathionine γ-lyase (CGL) [4], and by the detoxifying enzyme mercaptopyruvate sulfurtransferase [5]. The physiological targets vary according to the tissue, for example, in brain and nervous system, H_2_S can modulate NMDA receptors [2], whereas in the vasculature H_2_S mediates vasorelaxation by opening K_ATP_ channels [6]. It could also act as an oxidant scavenger, but the recent determination of the relatively low rate constants [7] together with the fairly low physiological levels [8], suggest that this action would depend on H_2_S being able to achieve high local concentrations.

If H_2_S were a hydrophobic molecule, then a higher local concentration of H_2_S could be achieved in the hydrophobic core of lipid membranes, and promote reactions with physiological targets. For instance, the reaction of nitric oxide with oxygen, which yields oxidizing and nitrosating species, occurs thirty times more rapidly inside lipid membranes than in aqueous media [9], [10], [11]. Considering that the permeability coefficient of a membrane to a solute (P_m_) is directly proportional to the partition coefficient (K_P_) [12], another consequence of a high solubility of H_2_S in lipid membranes would be a high membrane permeability. Mathai *et al.* have recently measured the permeability coefficient using planar lipid bilayers and found that diffusion through the membrane was fast indeed (P_m_ = 0.5±0.4 cm s^−1^, [13]). As discussed by these authors, this value may actually be an underestimation, which prompted us to find a better estimate. Diffusion of H_2_S through membranes and aqueous solution is very important because it will determine the extent of H_2_S action. If H_2_S could diffuse practically unhindered through lipid membranes, it could act at places distant from the site of formation supporting mechanisms of transient paracrine communication.

Understanding the interactions of H_2_S with lipid membranes and its overall diffusion is essential to rationalize the biological properties and the pharmacological potential of this newly recognized signaling molecule. Herein, we determined the partition coefficient of H_2_S in the organic solvents hexane and *n*-octanol, relative to water. We also developed a method and successfully measured the partition coefficient of H_2_S in dilauroylphosphatidylcholine liposome membranes. This solubility value allowed us to estimate the permeability coefficient of phospholipid membranes to H_2_S. Finally, we modeled the diffusional spread of H_2_S from a single cell, illustrating how far and how many neighboring cells could H_2_S affect, and analyzed the impact of lipid membranes on the macroscopic diffusion of H_2_S.

## Materials and Methods

### Hydrogen sulfide solutions

Stock solutions contained sodium hydrosulfide (NaHS, Sigma-Aldrich) in water and their concentration was determined by iodometric titration [7]. The working solutions contained a mix of H_2_S and HS^−^ (hydrosulfide anion) in dependence with pH (pK_a1_ = 7.0 and pK_a2_ ∼17 [14]). At pH 7.4 total sulfide distributes 72% as HS^−^, 28% as H_2_S and S^2−^ (sulfide) is insignificant. At pH 3.8 HS^−^ becomes negligible (0.06%) and solutions can be considered 100% H_2_S.

### Determination of partition coefficients in hexane and n-octanol

Partition coefficients (K_P_) were calculated as the ratio of organic solvent/buffer total sulfide concentration. Hexane and *n*-octanol were pre-equilibrated with sodium formate buffer (0.1 M, pH 3.8) overnight. Sealed tubes were prepared with a mix of buffer, sodium hydrosulfide (10 mM) and solvent with minimal headspace. Tubes were allowed to reach thermodynamical equilibrium (gentle agitation during 1 h at 25°C) and were centrifuged (10 min, 200 g) to separate the phases. Then, aliquots from both phases were removed with a gas-tight syringe and H_2_S was measured by an adaptation of the methylene blue assay [7], [15]. Samples taken at different times confirmed that the systems had reached equilibrium after one hour. No interference of the solvents in the quantification method was detected according to controls.

### Determination of the partition coefficient in DLPC liposomes

In the case of the dispersed liposomes, it is not possible to measure the concentration of H_2_S directly in the lipid phase, which does not separate from the aqueous phase. Thus, the partition coefficient was measured indirectly, according to the following reasoning: Consider two closed vials, one containing buffer only and the other a suspension of liposomes in buffer, both with a relatively large headspace. H_2_S will distribute in the three phases (gas, aqueous, lipid). If the same amount of H_2_S is added to both vials and H_2_S has a favorable partitioning in lipid membranes, it is expected that more H_2_S will be present in the liquid phase containing buffer and liposomes, than in the one containing buffer only. The inverse would happen if H_2_S had an unfavorable partitioning.

Large multilamellar liposomes were prepared by mechanical dispersion using dilauroylphosphatidylcholine (DLPC, Avanti Polar Lipids, 100 mg/mL) in formate buffer (0.1 M, pH 3.8). Septum-sealed vials (1980 µL) were prepared containing either liposomes in formate buffer or just formate buffer (100 µL), to which 20 mM H_2_S were added (10 µL) and allowed to reach equilibrium at 25°C (2 h, gentle agitation). Aliquots were withdrawn from the aqueous and the gaseous phase with a gas-tight syringe and H_2_S was measured by the methylene blue assay. Calibration curves included DLPC.

The method to determine K_P_ in membranes is based on the one previously used for nitric oxide (^·^NO) [16]. For H_2_S, we must consider the 3 phases to determine the K_P_ between membranes and water. Using mass conservation relationships, we could arrive to a simple expression, Equation 1 (the complete derivation of this equation is shown in Text S1) to calculate the partition coefficient, K_P_^mem/w^, the ratio of lipid/buffer H_2_S concentrations at equilibrium (25°C):
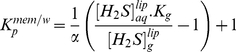
(1)where 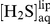 and 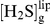 are aqueous and gas concentrations in the samples with liposomes, K_g_ is a partition-like expression of Henry's constant ([H_2_S]_g_/[H_2_S]_aq_), which can be calculated from the results obtained with buffer-only samples, and α is the lipid fractional volume, calculated taking into account lipid concentration and lipid specific volume (0.97 ml/g, see Text S1). Typical experimental concentration values are shown in Table S1. Measured K_g_ values, 0.4±0.1, were similar to previous reports [17].

### Modeling the 3D diffusional spread of H_2_S from a single cell

We wanted a mathematical model that could represent the diffusion of H_2_S in tissue. For that reason we chose a three dimensional model where the source is spherical and H_2_S diffuses from the surface. Furthermore, we chose a continuous source to better represent H_2_S cellular production. The corresponding solution of Fick's second law of diffusion is [18]:
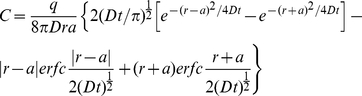
(2)where C is the concentration at a given distance r from the center of the sphere, *a* is the radius of the sphere, set to 5 µm, D is the diffusion coefficient of H_2_S, *q* is the rate of production of H_2_S and erfc is the complementary error function. *q* was set so that the concentration of H_2_S on the surface of the sphere would be 100.0 arbitrary units at infinite time (*q* = 1.46×10^7^) in aqueous media (without membrane resistance, D_w_ = 2.32×10^−5^ cm^2^ s^−1^).

## Results and Discussion

### Partitioning in organic solvents

Because of the difficulty involved in measuring the solubility of molecules in lipid membranes, organic solvents are usually used as surrogates. Octanol has been widely used for this purpose, and measuring a drug's partition coefficient in *n*-octanol is a common practice used to estimate the drug's biodistribution properties [19], [20]. At pH 3.8, where H_2_S predominates (pK_a_ = 7.0 [14]), the partition coefficient for H_2_S in *n*-octanol/buffer at 25°C resulted to be 2.1±0.2 (Table 1). Hexane was also used, as a completely non-polar solvent that could approximate the environment experienced by H_2_S in the mid-bilayer. The partition coefficient for H_2_S in hexane/buffer systems was 1.9±0.5 (Table 1). These values mean that H_2_S is twice as soluble in the organic solvents as in water. At the physiological pH of 7.4, the measured ratio was 0.64±0.05 for *n*-octanol. This lower apparent K_P_ is explained by the ionization of H_2_S to HS^−^ in the aqueous phase, which has a negligible solubility in the organic phase [21]. This is in full agreement with the calculated partition coefficient (K_P_^oc/w^ = 0.6 at pH 7.4) using K_P_^oc/w^ = 2.1 for H_2_S, mass balance and Henderson-Hasselbach equilibrium considerations (Equation 3). In addition, at a pH of 6.5, which approximates ischemic tissue acidosis, the apparent K_P_^oc/w^ can be calculated to be 1.6.
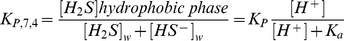
(3)


**Table 1:** Partition coefficients of H_2_S in membrane models.

Hydrophobic phase	pH	Partition coefficient, K_P_ a
Octanol^b^	3.8	2.1±0.2
Hexane^b^	3.8	1.9±0.5
DLPC liposomes^c^	3.8	2.0±0.6
Octanol^b^	7.4	0.64±0.05

a K_P_ was calculated as the ratio of sulfide concentrations in the hydrophobic phase/buffer phase at equilibrium at 25°C.

b The results are the average ± standard deviation of three independent experiments performed each in triplicates.

c The results are the average ± standard deviation of four independent experiments, performed each in quadruplicates.

### Partitioning in phospholipid membranes

Although K_P_^oc/w^ may already provide a valuable insight into the lipophilicity of H_2_S, considering that lipid membranes are intrinsically different from bulk solvent and constitute heterogeneous phases with high ordering, molecular packing and charge density [22], we proceeded to determine the partition coefficient of H_2_S between membranes of dilauroylphosphatidylcholine (DLPC) liposomes and water. According to our experimental data, the partition coefficient between phospholipid membranes and buffer was 2.0±0.6 (Table 1) at 25°C.

One potential consequence of this two-fold higher local concentration is the acceleration of reactions of H_2_S within the membrane. H_2_S is known to inhibit mitochondrial respiration by reacting with cytochrome *c* oxidase [23]. This is a transmembrane protein complex that has many of its metallic prosthetic groups located deep in the transmembrane domain. It is very likely that the two-fold higher concentration of H_2_S in the hydrophobic core of the membrane plays a role in facilitating the reaction of H_2_S with these metallic centers and inhibiting its activity.

We mentioned earlier that the hydrophobicity of H_2_S could enhance its antioxidant potential in lipid membranes where low molecular weight thiols such as glutathione are scarce. This was an interesting possibility, but there is a problem. We have recently shown that most of the reactions ascribed to H_2_S such as disulfide reduction, nucleophilic substitution and free radical scavenging are actually done by HS^−^, which is a better nucleophile, more reactive and is present in higher amounts at physiological pH [7]. The dissociation of H_2_S to HS^−^ in a lipid environment is thermodynamically unfavorable, so that, paradoxically, the net effect in lipid membranes should be a decrease in reactivity despite the favorable partitioning of H_2_S.

Another important consequence of the higher solubility of H_2_S in membranes than in water is a high membrane permeability, as will be discussed below.

### Estimation of the diffusion of H_2_S through lipid membranes

A recent work by Mathai *et al*. using planar lipid bilayers indicated that transport of H_2_S through biological membranes is indeed extremely fast [13]. In their report, a free-standing bilayer lipid membrane made of *E. coli* total lipid extract was used and measurements were made with microelectrodes near the membrane, assuming a steady-state approach. A lower limit for H_2_S permeability of 0.5±0.4 cm s^−1^ was reported. However, it was observed that addition of cholesterol and sphingomyelin to *E. coli* lipid membranes, which cause bilayer tightening and generally lead to a decrease in membrane permeability, had no effect on the measured P_m_, indicating that unstirred layer effects were very important and that the determined P_m_ is very likely an underestimation [13]. We tried to obtain better estimates through different approaches. Experimentally, we used stopped-flow to monitor H_2_S entrance into phospholipid liposomes, where we confirmed a very fast H_2_S permeation, in fact too fast to be measured (see Figure S1 and Text S2 for details). In a semi-theoretical approach, we used membrane permeability data for similar molecules to estimate the permeability coefficient of H_2_S.

According to the current view of the permeation process, one of the main factors controlling permeability is the solubility of the molecule in the membrane [24]. The permeability coefficient of a membrane is proportional to K_P_ and the diffusion coefficient in the membrane (D_m_), and inversely proportional to the width of the bilayer (dx in Equation 4) [24].
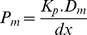
(4)


Partition coefficients found here (Table 1) suggested a permeability coefficient for H_2_S higher than reported. The permeability of lipid bilayers to molecules comparable to H_2_S, such as hydrogen chloride or carbon dioxide, is high: 2.9 and >3.2 cm s^−1^, respectively. Considering the molecular volume, water solubility and partition coefficients (Table 2) we would then expect a permeability coefficient of H_2_S in lipid bilayers equal to or higher than 3 cm s^−1^. Note that ^·^NO and O_2_ may not be the best models for H_2_S behavior given their low solubility in water and larger K_P_^oc/w^ (Table 2).

**Table 2:** Partition and permeability coefficients of gaseous molecules (at 25°C).

	Dipole moment (D)	Molecular volume (Å^3^)^a^	Solubility in water (mM/atm)^b^	Partition coefficient K_P_^oc/w^	Partition coefficient K_P_^mem/w^	Permeability coefficient P_m_ (cm s^−1^)
H_2_S	0.97	29.24	100	2.1±0.2^c^	2.0±0.6^c^	>0.5 [13] – 3^d^
HCl	1.11	25.07	1.9×10^4^	1.8 [24]	ND	2.9 [32]
CO_2_	0	34.26	34	1.3 [33]	0.95 [33]	>3.2 [34]
^·^NO	0.159	23.70	1.95	6.5 [35]	3.6 [10]	93 [36]
O_2_	0	23.18	1.3	5.6 [37]	3.2 [10]	67 [36]

a Calculated using Molinspiration [38].

b Data obtained from [17], [39].

c Determined in this work.

d Estimated in this work.

Taking the value of 3 cm s^−1^ for the permeability coefficient and with the partition value for membranes of 2.0 determined herein, we can estimate a diffusion coefficient of 6×10^−7^ cm^2^ s^−1^ for H_2_S in lipid membranes (D_m_ in Equation 4). This value is significantly lower than the diffusion coefficient in water, D_w_ = 2.32×10^−5^ cm^2^ s^−1^ at 35°C [25]. So, are lipid membranes effective barriers to H_2_S transport? We can easily calculate the resistance to H_2_S flux imposed in a cell by lipid membranes. If all sulfide consisted of H_2_S, as at acidic pHs, considering P_m_ = 3 cm s^−1^, the resistance of one 4 nm-thick (dx) phospholipid membrane would be 1/P_m_ = 0.33 cm^−1^ s. The resistance of an equally thick layer of water would be 1/P_w_ = 0.017 cm^−1^ s (using a permeability of 58 cm s^−1^, calculated as P_w_ = D_w_/dx, analogous to Equation 4 [25]). Several membranes would behave as resistances in series [24], so that considering the contribution of several 4 nm-thick layers of water (*a–n*) and membranes (*n*), a weighed apparent total resistance (1/P_T_) for the whole process can be calculated using Equation 5:
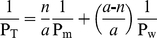
(5)Assuming that simple diffusion in a cell of 10 µm in diameter equals diffusion in *a* = 2500 layers, 4 nm each, of water, it can be calculated that a single membrane (*n* = 1) would result in a very small decrease in diffusion (0.7%). Even assuming that H_2_S must diffuse across several organelle membranes accounting for 20 lipid bilayers (*n* = 20), a total decrease in diffusion of only 12.8% can be calculated. In this acidic pH situation, the resistance would be low and lipid membranes would not limit considerably the diffusion of H_2_S.

However, this scenario changes significantly if we consider that, at the physiological pH of 7.4, a high proportion of total sulfide is present as HS^−^ (72%) instead of H_2_S (28%). Both H_2_S and HS^−^ diffuse at similar rates in the aqueous layers, whereas in membrane layers, H_2_S diffuses, while the HS^−^ anion does not diffuse at all. The apparent membrane permeability for the H_2_S and HS^−^ pair found at pH 7.4 in physiological conditions would be P_m, 7.4_ = 0.85 cm s^−1^ (calculated using Equation 6). Using this corrected value of P_m_ at pH 7.4, the net effect of the 20 lipid membranes in the cell would be a decrease of 34.9% in the apparent diffusion.
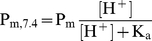
(6)


Lowering the pH would result in an increase in apparent diffusion. In ischemia, for instance, there is tissue acidosis, and the pH can decrease to 6.5. At this pH, the apparent P_m_ would be 2.3 cm s^−1^ and the apparent diffusion would decrease 16.4% in the presence of membranes.

Overall, lipid membranes will offer a low resistance to the diffusion of H_2_S that will not limit its transport across cells to a great extent. The effect of multiple cells and lipid membranes (“tissue”) on H_2_S macroscopic diffusion is discussed next.

### Modeling the diffusional spread of H_2_S from a single cell

**Figure 1:**
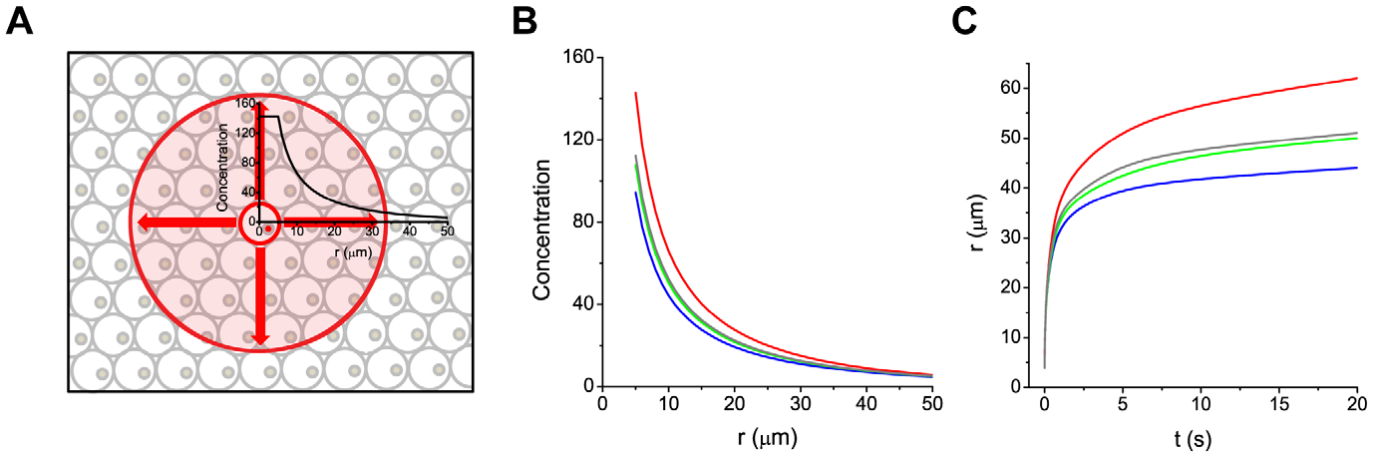
Modeling three-dimensional diffusion from a single cell. **A**) The model consists of a single spherical cell producing H_2_S at a constant rate. We interrogate how the concentration of total sulfide (H_2_S+HS^−^) changes as a function of time and distance from the source cell with or without surrounding cells. The sphere of action is defined by the distance from the source cell at which the concentration of total sulfide is 10.0 arbitrary units. **B**) Expansion 1s after formation starts, with membranes at acidic pH (green), with membranes at pH 7.4 (red), with membranes at pH 6.5 (gray) and without membrane resistance (blue) **C**) Expansion of the sphere of action as a function of time, without membrane resistance (blue), with membranes at acidic pH (green), with membranes at pH 7.4 (red) and at pH 6.5 (gray). Plots are derived from Equation 2. The resistance imposed by the membranes was weighed into the aqueous diffusion coefficient (Equation 5) so that 20 membranes would cause the apparent diffusion coefficient of H_2_S to decrease from D_w_ = 2.32×10^−5^ cm^2^ s^−1^ (H_2_S in water, blue line) to 2.02×10^−5^ cm^2^ s^−1^ (green line). Considering ionization to HS^−^, the apparent diffusion coefficient of H_2_S/HS^−^ drops to 1.51×10^−5^ cm^2^ s^−1^ at pH 7.4 (red line) and to 1.94×10^−5^ cm^2^ s^−1^ at pH 6.5 (gray line).

The relatively low barrier to transport offered by lipid membranes indicates that H_2_S produced in one cell can diffuse and exert effects on distant cells, complying with the requirements of a paracrine signaling molecule. Although it has often been compared with ^·^NO and CO [26], no attempt to model H_2_S diffusion in tissues has been made. In contrast to early one-dimensional point-source models of ^·^NO diffusion [27], [28] here we used a 3D diffusion model involving a spherical source (“the cell”) that produces H_2_S in a continuous manner from the surface. By using a spherical model, we include many sources of H_2_S (collection of enzymes) while avoiding the complexities derived of trying to use multiple single-point sources in a 3D model. We can calculate the change in the concentration of H_2_S as a function of time and distance from the source. Since there is still some debate about how much H_2_S is generated by cells and how much is necessary to activate different functions, we used a generic model where we set the rate of H_2_S production so that the concentration of H_2_S at the surface of the cell is 100.0 arbitrary units at infinite time in the absence of membrane resistance. The sphere of action was arbitrarily set at the distance in which the concentration of H_2_S drops to 10.0. Another reason to use a generic model with arbitrary units is that it can be conveniently scaled to any concentration. If we knew the actual concentration of H_2_S in the surface of a cell, we could rescale Figure 1B directly, and obtain the actual concentration distribution of H_2_S away from a cell.

Considering the free diffusion of H_2_S in an aqueous medium with no membranes, we can see that after production starts, the sphere of action expands very rapidly and has a radius of 32 µm after 1 s and 42 µm after 10 s (Figure 1C). By calculating how many spheres of 10 µm diameter (representing cells) can fit within spheres of 32 and 42 µm radii, we can estimate that H_2_S would be able to reach 260 and 590 neighboring cells, respectively. After incorporating the resistance imposed by lipid membranes to H_2_S (n = 20, D_T_ = 2.02×10^−5^ cm^2^ s^−1^), we found that, for the same rate of production, the concentration close to the cell was significantly increased (Figure 1B). At pH 7.4, where ionization to HS^−^ decreases the apparent membrane permeability (D_T, 7.4_ = 1.51×10^−5^ cm^2^ s^−1^), the increase in concentration was even more remarkable. For example, at 20 µm from the surface of the cell after 1 s of production, the concentration of sulfide was 40% higher than in the absence of membranes. By slowing down diffusion, total sulfide spreads more slowly and a higher concentration is achieved close to the source. As defined here, the sphere of action in absolute numbers is actually larger for the hindered diffusion model (Figure 1C), but expectedly decreases when the concentration at the source is re-normalized to 100.0. This effect of hindered diffusion may help to build up a higher local concentration of H_2_S, focusing the signaling function close to the site of H_2_S production (see also Figure S2). At the pH of 6.5 typical of tissue acidosis, the focusing effect of the membranes was lower than at pH 7.4 (Figure 1B), consistent with a higher proportion of H_2_S being protonated at this pH (D_T, 6.5_ = 1.94×10^−5^ cm^2^ s^−1^).

The size of the sphere of action will also depend on how fast H_2_S is consumed as it spreads in a tissue. The decay of H_2_S will be determined by the presence of molecular targets like disulfide bonds, oxidants, mitochondrial membrane proteins and metallic centers among others, and on how fast H_2_S reacts with these targets. In rat blood, for instance, the half-life of sulfide has been reported to be 151 s [29]. Considering that in 15 seconds H_2_S covers 90% of the space covered at infinite time (Figure 1C), while only ∼7% of the starting H_2_S has been consumed, such a rate of decay will have minimal effects on H_2_S spread.

In summary, H_2_S produced at one site should easily reach proximal cell layers at concentrations close to those in the source (Figure 1A–C), and the layers further away at decreasing concentrations, supporting the role of hydrogen sulfide as a paracrine signaling molecule. More studies on the rate and concentration of H_2_S that can be produced by cells in different tissues are needed to define the real extent and range of physiological effects of H_2_S.

### H_2_S diffusion and partitioning in ischemia-reperfusion

It has been shown that exogenous H_2_S can protect cardiac muscle cells from ischemia-reperfusion injury when it is added during reperfusion, reducing significantly the infarct size and subsequent inflammation [30], [31]. In a study, exogenous H_2_S reduced the infarct size by 70% in the hearts of mice subjected to ischemia-reperfusion [30]. This high degree of myocardial protection is likely due to the high membrane permeability and diffusivity of H_2_S, further enhanced by tissue acidosis that allows H_2_S to go deep in the myocardium. Partitioning of H_2_S in the mitochondrial membranes may also be involved in protecting against ischemia-reperfusion injury, since part of the protective actions of H_2_S is ascribed to the inhibition of cytochrome c oxidase [30]. As discussed earlier, the reaction of H_2_S with this protein complex is likely enhanced by the two-fold higher solubility of H_2_S in membranes relative to water. The high membrane permeability and partitioning of H_2_S are undoubtedly very advantageous pharmacokinetic properties.

### Conclusions

We found that H_2_S is twice as soluble in lipid membranes as in water (K_P_ = 2.0±0.6), and similar results were found with *n*-octanol and hexane (K_P_ = 2.1 and 1.9, respectively). The estimated high membrane permeability coefficient of H_2_S (3 cm s^−1^) indicates a very low barrier to intercellular transport. A 3D mathematical model of H_2_S diffusion in tissues at pH 7.4 shows that the low but significant resistance imposed by lipid membranes slows down diffusion in tissues and leads to a local accumulation of H_2_S near the source. In these conditions, the sphere of action, defined by the distance at which the concentration of H_2_S is 10% that at the source, involves more than 200 neighboring cells within 1 s of formation. These results support the role of hydrogen sulfide as a paracrine signaling molecule and reveal advantageous pharmacokinetic properties for its therapeutic applications.

## Supporting Information

Text S1 **Derivation of** **Equation 1** **.**
(DOC)

Text S2 **Permeation in liposomes.** Experimental details and equations.
(DOC)

Figure S1 **Permeation of hydrogen sulfide through liposome phospholipid membranes.** **A**) Reaction scheme showing the transport of protons across the membrane by H_2_S, leading to intravesicular acidification. **B**) Decrease in intravesicular HPTS fluorescence caused by H_2_S, indicating entrance of H_2_S into the vesicle and intravesicular acidification. HPTS is shown in the inset. Fluorescence was measured 30 seconds after adding H_2_S. **C**) Stopped-flow profile for H_2_S entrance. The entrance was nearly complete within the first second of measurement. **D**) Stopped-flow profile for acetic acid entrance, showing a well defined change in fluorescence. For all experiments, HPTS (2 mM) was encapsulated in DMPC∶cholesterol 1∶1 unilamellar liposomes in Tris buffer (10 mM, KCl 150 mM, pH 8.0). Fluorescence emission was measured at 510 nm (λ_ex_ = 454 nm). H_2_S was introduced as a NaHS solution. The concentrations of NaHS and acetic acid used in these experiments are indicated in the figures.
(TIF)

Figure S2 **Diffusional spread dependence on membrane resistance.** **A**) Concentration profiles of total sulfide (H_2_S+HS^−^) as a function of time and distance considering a spherical and continuous source (“cell”) with unhindered diffusion (r = 5 µm, D = 2.32×10^−5^ cm^2^ s^−1^); **B**) with a resistance of 20 membranes per cell (D = 2.02×10^−5^ cm^2^ s^−1^); and **C**) with a resistance of 20 membranes per cell at pH 7.4 (D = 1.51×10^−5^ cm^2^ s^−1^). The gray line at 10.0 concentration units indicates the limit of the putative sphere of action. As better exemplified in C, an important consequence of slowing down diffusion is increasing the concentration of H_2_S near the origin and throughout the system. The spreading does occur more slowly, but a higher concentration can be achieved near the source. Concentration profiles were calculated using Equation 2 and expressed as arbitrary units.
(TIF)

Table S1 **Typical experimental values used to calculate K_P_^mem/w^ in liposomes.**
(DOC)
