# Supplementary material for: Solubility and Permeation of Hydrogen Sulfide in Lipid Membranes
Source: PLoS One. 2012 Apr 11;7(4):e34562. doi: 10.1371/journal.pone.0034562 (PMC3324494; doi:10.1371/journal.pone.0034562)
Supplement: Text S1 — Derivation of Equation 1 . (DOC) [file pone.0034562.s001.doc]

**Text S1. *Derivation of Equation 1***

Consider a closed system, with gas (g) and aqueous phase (aq).

$C_{T}.V_{T}=C_{g}.V_{g}+C_{aq}.V_{aq}$ Eq. S1

When the aqueous phase contains a heterogeneous lipid phase, we can separate it in buffer (w) and lipid phases (l).

$C_{T}.V_{T}=C_{g}.V_{g}+C_{w}.V_{w}+C_{l}.V_{l}$ Eq. S2

Defining the Henry constant analog K_g_, and the partition ratio K_P_,

$K_{g}=\frac{C_{g}}{C_{w}}$ Eq. S3

$K_{P}=\frac{C_{l}}{C_{w}}$ Eq. S4

and replacing in Eq. S2,

$C_{T}.V_{T}=C_{g}.V_{g}+\frac{C_{g}}{K_{g}}.V_{w}+K_{P}\frac{C_{g}}{K_{g}}.V_{l}$ Eq. S5

we can go back to the definition in Eq. S1

$C_{g}.V_{g}+C_{aq}.V_{aq}=C_{g}.V_{g}+\frac{C_{g}}{K_{g}}.V_{w}+K_{P}\frac{C_{g}}{K_{g}}.V_{l}$ Eq. S6

and eliminate part of the gas terms.

$C_{aq}.V_{aq}=\frac{C_{g}}{K_{g}}.V_{w}+K_{P}\frac{C_{g}}{K_{g}}.V_{l}$ Eq. S7

Rearranging this equation, we can get K_P_.

$K_{P}=\frac{C_{aq}.V_{aq}.K_{g}}{C_{g}.V_{l}}-\frac{V_{w}}{V_{l}}$ Eq. S8

Defining then the fractional lipid volume as in Eq. S9,

$\alpha\equiv\frac{V_{l}}{V_{aq}}=\frac{V_{l}}{V_{l}+V_{w}}$ Eq. S9

$V_{w}=V_{l}.\frac{\left(1-\alpha\right)}{\alpha}$ Eq. S10

we also get Eq. S10 and replacing in Eq. S8 yields:

$K_{P}=\frac{C_{aq}.K_{g}}{C_{g}.\alpha}-\frac{1}{\alpha}+1$ Eq. S11

Rearranging, we get Equation 1, as used in the main text.

$K_{P}^{mem/w}=\frac{1}{\alpha}.\left(\frac{C_{aq}.K_{g}}{C_{g}}-1\right)+1$ **Eq. 1**

Note that α can be calculated from the lipid mass and specific volume (*v*):

$\alpha\equiv\frac{V_{l}}{V_{aq}}=\frac{m_{l}.v}{V_{aq}}$ Eq. S12
