# Supplementary material for: Solubility and Permeation of Hydrogen Sulfide in Lipid Membranes
Source: PLoS One. 2012 Apr 11;7(4):e34562. doi: 10.1371/journal.pone.0034562 (PMC3324494; doi:10.1371/journal.pone.0034562)
Supplement: Text S2 — Permeation in liposomes. Experimental details and equations. (DOC) [file pone.0034562.s002.doc]

**Text S2. Permeation in liposomes**

***Experimental details***

In order to measure permeation through liposome membranes, we decided to use a fluorescent pH probe encapsulated in unilamellar liposomes. As H_2_S permeates through the membrane and into the intravesicular space, it dissociates releasing protons and acidifying the interior of the liposome. This leads to a decrease in probe emission which can be followed by fluorescence spectroscopy [1]. The time course of fluorescence decrease should reflect the rate of entrance since protonation of the probe is controlled by diffusion.

Encapsulation of the probe in good yields was achieved with 8-hydroxypyrene-1,3,6-trisulfonate (HPTS, Sigma-Aldrich) and dimyristoyl-phosphatidylcholine (DMPC): cholesterol 1:1 (Avanti Polar Lipids) unilamellar liposomes, which membranes are in liquid-ordered phase at 25°C [2]. Briefly, HPTS (2mM) in Tris buffer (10 mM, KCl 150 mM, pH 8.0) was added to dried lipids (20 mg/ml after mixing). Following hydration and liposome formation through shaking, the preparation was extruded through a 100 nm pore-size filter in order to homogenize the radius of the liposomes to 60-70 nm [3]. Then, liposomes were passed through a size exclusion column (PD-10, GE) to remove unincorporated HPTS and were eluted with Tris buffer (10 mM, KCl 150 mM, pH 8.0). H_2_S and acetic acid solutions were diluted in the same buffer in which liposomes were prepared. Kinetic measurements were made in an AMINCO-Bowman Series 2 fluorimeter connected to a stopped-flow device (Applied Photophysics RX2000). Fluorescence was followed by excitation at 454 nm and emission at 510 nm.

To calculate the permeability coefficient, we used the model reported by Faure *et al.* [4], modified for a weak acid entrance according to Xiang *et al.* [5], arriving at Equation S13 (derived below).

$\text{P}_{\text{m}}\text{ = }\frac{\text{k}_{\text{obs}}\text{.V}_{\text{in}}}{\text{S}}.\frac{\text{[H}^{\text{+}}\text{] + K}_{\text{a}}}{\text{[H}^{\text{+}}\text{]}}\text{= P}_{\text{m, obs}}.\frac{\text{[H}^{\text{+}}\text{] + K}_{\text{a}}}{\text{[H}^{\text{+}}\text{]}}$ Eq. S13

To determine k_obs_, fluorescence decays were fitted to single exponential functions. Surface area (S = 53.1 x 10^3^ nm^2^) and interior volume (V_in_ = 1.15 x 10^6^ nm^3^) were calculated from the liposome radius (r = 65 nm). K_a_ is the acid constant for the permeant weak acid (1.00 x 10^-7^ for H_2_S and 1.74 x 10^-5^ for acetic acid).

***Permeation Results***

Experiments with the fluorescence probe HPTS, which senses pH changes near physiological conditions (pK_a_ = 7.25), demonstrated that H_2_S permeates through the liposome membranes. The decrease in fluorescence emission indicated that the internal medium was acidified and confirmed no significant liposome lysis or HPTS leakage (since the external solution was alkalinized by basic NaHS additions) (Figure S1A and B).

Controls were done with acetic acid (Figure S1D), where good kinetic traces could be obtained and the observed permeability coefficient was measured as (2.6 ± 0.4) x 10^-4^ cm s^-1^, similar to previous reports [6]. In contrast, the entrance of H_2_S occurred before the first observation times, confirming that permeation is indeed fast (Figure S1C), so fast that it could not be observed under our experimental conditions. As an alternative, we tried to measure the diffusion coefficient of H_2_S in membranes using fluorescence quenching in order to estimate the permeability coefficient [7,8]. Unfortunately, no diffusional quenching by H_2_S was observed with the typical probes pyrenecarboxylic acid nor tryptophan.

***Derivation of equation S13***

Consider a weak acid that can exist in protonated (HA) and unprotonated (A^-^) states in equilibrium. Only HA can traverse the membrane. Using the Fick model for diffusion in a monolamellar liposome:

$\text{J}_{\text{weak acid}}\text{ = }\frac{\text{dn}_{\text{weak acid}_{\text{T}}}}{\text{dt.S}}\text{ = P}_{\text{m}}\text{ . Δ[HA]}$ Eq. S14

where S is the superficial area of the liposome and P the permeability coefficient.

$\frac{\text{d[weak acid]}_{\text{T}}}{\text{dt}}\text{ . }\frac{\text{V}_{\text{in}}}{\text{S}}\text{ = P}_{\text{m}}\text{ ([HA]}_{\text{out}}\text{ - [HA]}_{\text{in}}\text{)}$ Eq. S15

where V_in_ is the internal volume of the liposome

$\frac{\text{d[weak acid]}_{\text{T}}}{\text{dt}}\text{ = }\frac{\text{P}_{\text{m}}\text{.S}}{\text{V}_{\text{in}}}\text{ ([HA]}_{\text{out}}\text{ - [HA]}_{\text{in}}\text{)}$ Eq. S16

and using mass balance:

$\text{K}_{\text{a}}\text{ = }\frac{\text{[H}^{\text{+}}\text{].[A}^{\text{-}}\text{]}}{\text{[HA]}}\text{, [weak acid]}_{\text{T}}\text{ = [HA] + [A}^{\text{-}}\text{]}$ Eq. S17

$\text{[weak acid]}_{\text{T}}\text{ = [HA] }\frac{\text{[H}^{\text{+}}\text{] + K}_{\text{a}}}{\text{[H}^{\text{+}}\text{]}}$ Eq. S18

we can arrive to:

$\frac{\text{d[HA]}_{\text{in}}}{\text{dt}}\text{ . }\frac{\text{[H}^{\text{+}}\text{]}_{\text{in}}\text{ + K}_{\text{a}}}{\text{[H}^{\text{+}}\text{]}_{\text{in}}}\text{ = }\frac{\text{P}_{\text{m}}\text{.S}}{\text{V}_{\text{in}}}\text{ ([HA]}_{\text{out}}\text{ - [HA]}_{\text{in}}\text{)}$ Eq. S19

$\frac{\text{d[HA]}_{\text{in}}}{\text{[HA]}_{\text{out}}\text{ - [HA]}_{\text{in}}}\text{ = }\frac{\text{P}_{\text{m}}\text{.S}}{\text{V}_{\text{in}}}\text{ . }\frac{\text{[H}^{\text{+}}\text{]}_{\text{in}}}{\text{[H}^{\text{+}}\text{]}_{\text{in}}\text{ + K}_{\text{a}}}\text{ dt}$ Eq. S20

Integrating as function of [HA]_in_, considering that at t = 0, [HA]_in_ = 0 and assuming pH_in_ constant:

$\text{[HA]}_{\text{in}}\text{ = [HA]}_{\text{out}}\text{ (1 - e}^{\text{-k}_{\text{obs}}\text{.t}}\text{), k}_{\text{obs}}\text{ = }\frac{\text{P}_{\text{m}}\text{.S}}{\text{V}_{\text{in}}}\text{ . }\frac{\text{[H}^{\text{+}}\text{]}_{\text{in}}}{\text{[H}^{\text{+}}\text{]}_{\text{in}}\text{ + K}_{\text{a}}}$ Eq. S21

Rearranging, we get Equation S13:

$\text{P}_{\text{m}}\text{ = }\frac{\text{k}_{\text{obs}}\text{.V}_{\text{in}}}{\text{S}}\text{ . }\frac{\text{[H}^{\text{+}}\text{]}_{\text{in}}\text{ + K}_{\text{a}}}{\text{[H}^{\text{+}}\text{]}_{\text{in}}}$ **Eq. S13**

**References**

1. Lande MB, Donovan JM, Zeidel ML (1995) The relationship between membrane fluidity and permeabilities to water, solutes, ammonia, and protons. J Gen Physiol 106: 67-84.

2. Almeida PF, Vaz WL, Thompson TE (1992) Lateral diffusion in the liquid phases of dimyristoylphosphatidylcholine/cholesterol lipid bilayers: a free volume analysis. Biochemistry 31: 6739-6747.

3. Nayar R, Hope MJ, Cullis PR (1989) Generation of large unilamellar vesicles from long-chain saturated phosphatidylcholines by extrusion technique. Biochim Biophys Acta 986: 200-206.

4. Faure C, Nallet F, Roux D, Milner ST, Gauffre F, et al. (2006) Modeling leakage kinetics from multilamellar vesicles for membrane permeability determination: application to glucose. Biophys J 91: 4340-4349.

5. Xiang TX, Anderson BD (1998) Influence of chain ordering on the selectivity of dipalmitoylphosphatidylcholine bilayer membranes for permeant size and shape. Biophys J 75: 2658-2671.

6. Xiang TX, Anderson BD (1997) Permeability of acetic acid across gel and liquid-crystalline lipid bilayers conforms to free-surface-area theory. Biophys J 72: 223-237.

7. Denicola A, Batthyany C, Lissi E, Freeman BA, Rubbo H, et al. (2002) Diffusion of nitric oxide into low density lipoprotein. J Biol Chem 277: 932-936.

8. Denicola A, Souza JM, Radi R, Lissi E (1996) Nitric oxide diffusion in membranes determined by fluorescence quenching. Arch Biochem Biophys 328: 208-212.
