# Supplementary figures and images for: Solubility and Permeation of Hydrogen Sulfide in Lipid Membranes
Source: PLoS One. 2012 Apr 11;7(4):e34562. doi: 10.1371/journal.pone.0034562 (PMC3324494; doi:10.1371/journal.pone.0034562)

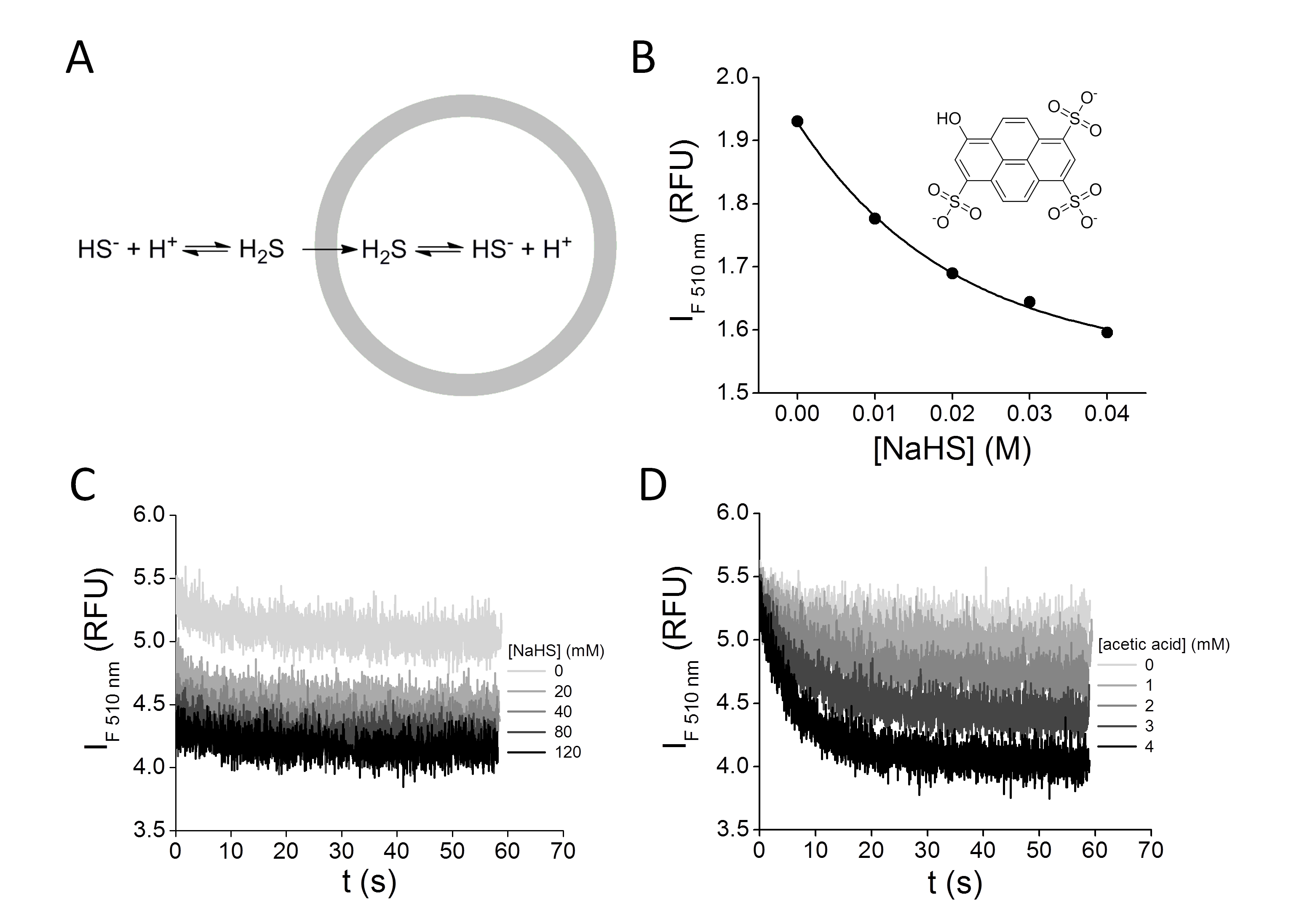

Supplement: Figure S1 — Permeation of hydrogen sulfide through liposome phospholipid membranes. A) Reaction scheme showing the transport of protons across the membrane by H2S, leading to intravesicular acidification. B) Decrease in intravesicular HPTS fluorescence caused by H2S, indicating entrance of H2S into the vesicle and intravesicular acidification. HPTS is shown in the inset. Fluorescence was measured 30 seconds after adding H2S. C) Stopped-flow profile for H2S entrance. The entrance was nearly complete within the first second of measurement. D) Stopped-flow profile for acetic acid entrance, showing a well defined change in fluorescence. For all experiments, HPTS (2 mM) was encapsulated in DMPC∶cholesterol 1∶1 unilamellar liposomes in Tris buffer (10 mM, KCl 150 mM, pH 8.0). Fluorescence emission was measured at 510 nm (λex = 454 nm). H2S was introduced as a NaHS solution. The concentrations of NaHS and acetic acid used in these experiments are indicated in the figures. (TIF) [file pone.0034562.s003.tif]

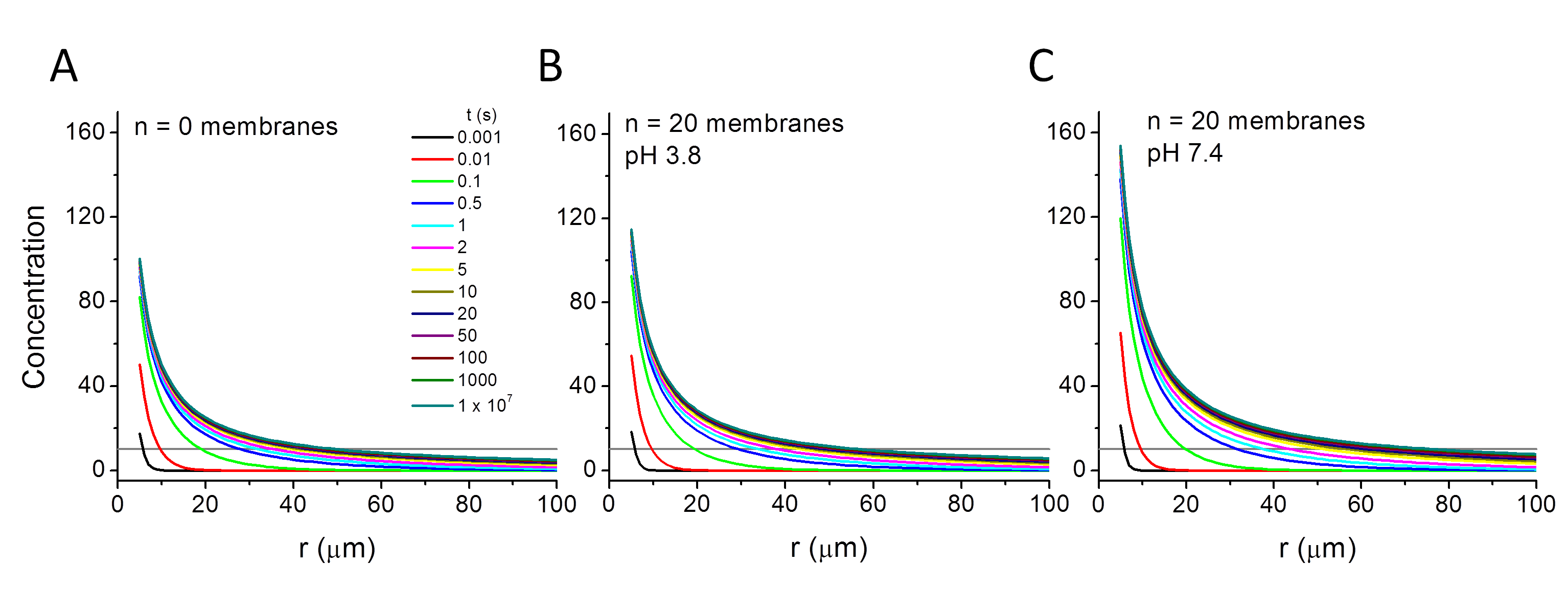

Supplement: Figure S2 — Diffusional spread dependence on membrane resistance. A) Concentration profiles of total sulfide (H2S+HS−) as a function of time and distance considering a spherical and continuous source (“cell”) with unhindered diffusion (r = 5 µm, D = 2.32×10^−5^ cm2 s−1); B) with a resistance of 20 membranes per cell (D = 2.02×10^−5^ cm2 s−1); and C) with a resistance of 20 membranes per cell at pH 7.4 (D = 1.51×10^−5^ cm2 s−1). The gray line at 10.0 concentration units indicates the limit of the putative sphere of action. As better exemplified in C, an important consequence of slowing down diffusion is increasing the concentration of H2S near the origin and throughout the system. The spreading does occur more slowly, but a higher concentration can be achieved near the source. Concentration profiles were calculated using Equation 2 and expressed as arbitrary units. (TIF) [file pone.0034562.s004.tif]
