# Supplementary material for: Solubility and Permeation of Hydrogen Sulfide in Lipid Membranes
Source: PLoS One. 2012 Apr 11;7(4):e34562. doi: 10.1371/journal.pone.0034562 (PMC3324494; doi:10.1371/journal.pone.0034562)
Supplement: Table S1 — Typical experimental values used to calculate KPmem/w in liposomes. (DOC) [file pone.0034562.s005.doc]

**Table S1. Typical experimental values used to calculate K_P_^mem/w^ in liposomes.**

| **Sample** | **Buffer-only** | **Buffer and liposomes** |
| --- | --- | --- |
| **[H_2_S]_g_ (mM)** | 0.87 | 0.85 |
| **[H_2_S]_aq_ (mM)** | 2.07 | 2.23 |

The concentration of H_2_S was measured both in the gas and aqueous phase of sample vials after 2 hours incubation at 25^o^C. The vials were gas-tight (1980 μL) and contained 100 μL buffer (0.1 M sodium formate, pH 3.8) or 100 μL DLPC liposomes (91 mg/ml) in the same buffer. In this experiment, α was 0.091, K_g_ was 0.42, and K_P_^mem/w^, calculated as described by Eq. 1, was 2.14.
